# Valorization of *Pistacia vera* By‐Products: Monthly Variation of Bioactive Compounds and Biological Activities

**DOI:** 10.1002/cbdv.71846

**Published:** 2026-10-27

**Authors:** Fatma Dagli, Nazan Comlekcioglu

**Affiliations:** ^1^ Department of Biology Faculty of Science Kahramanmaras Sutcu Imam University Kahramanmaras Turkiye

**Keywords:** agricultural waste valorization, bioactive compounds, fatty acid composition, monthly variation, *Pistacia vera*

## Abstract

*Pistacia vera* (*P. vera*) by‐products, particularly leaves and fruit hulls, are largely discarded during processing despite their phytochemical richness. This study investigated monthly variations in phenolic and flavonoid contents, fatty acid composition, antioxidant capacity, and antimicrobial activity in *P. vera* leaves and fruit hulls collected from May to November. Both tissues were rich in phenolic and flavonoid compounds, with the highest levels observed in spring. Antioxidant activity, assessed by DPPH and FRAP assays, followed a similar monthly pattern. Polyunsaturated fatty acids, mainly linoleic and α‐linolenic acids, predominated in both tissues. Fruit hulls exhibited greater monthly variation in linoleic acid than leaves. In hulls, oleic acid was predominantly negatively associated with antioxidant parameters, whereas linoleic acid showed positive associations; these tissue‐specific patterns are considered preliminary observations. Inhibition zones reached 28–31 mm against *Serratia marcescens* and *Micrococcus luteus*, with MIC values of 8–32 mg/mL. Leaf extracts showed higher antibacterial activity than hull extracts. These findings highlight *P. vera* by‐products as potential sources of bioactive compounds and provide preliminary information for identifying favorable harvest periods, although industrial feasibility was not evaluated.

## Graphical abstract

*Pistacia vera* leaves and hulls, generally discarded as processing waste, are profiled monthly for phenolic, flavonoid, fatty acid, antioxidant, and antimicrobial properties. Phenolic content, flavonoid content, and antioxidant activity peak in spring, while polyunsaturated fatty acids predominate in both tissues. Leaf and hull extracts inhibit key bacterial strains, highlighting these by‐products as promising, season‐dependent sources of natural bioactive compounds.

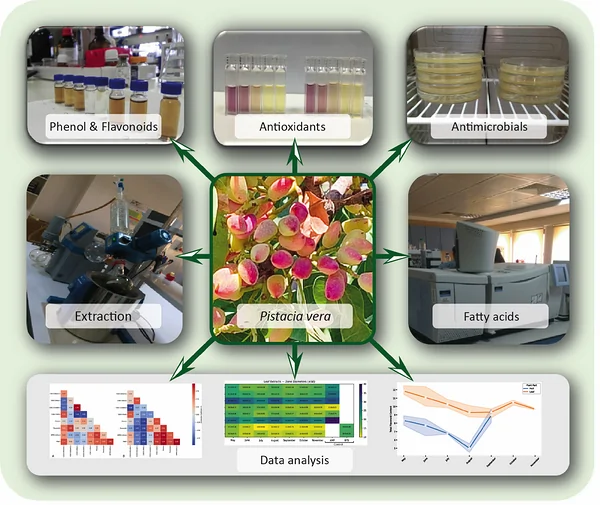

## 1 Introduction

*Pistacia vera* (*P. vera*) L. (pistachio) is a member of the *Anacardiaceae* family and an economically valuable species that grows in the arid regions of Central and Western Asia [1]. During the 2024–2025 marketing year, global pistachio production was reported to reach approximately 1.18 million tons, with the majority originating from the United States, Turkey, Iran, and Syria. In the same season, Turkey entered a high‐yield “on‐year” production cycle, achieving a harvest of 385 000 tons and securing second place globally with a 33% share, following the United States [2]. The pistachio fruit consists of a single edible seed (kernel) surrounded by a thin testa and enclosed within a woody endocarp. The outer portion of the fruit includes two fleshy layers, the epicarp and mesocarp, which form the soft hull. During ripening, the hull changes color from green to yellowish‐red and eventually to pale pink‐yellow, becoming easily separable from the nut [3, 4]. During processing, this soft hull, which accounts for approximately 18% of the total fruit mass, is removed as waste after the kernel is extracted. The hull thus represents one of the most voluminous waste fractions generated by the pistachio processing industry [5]. Other by‐products, including leaves, clusters, woody shells, and residual kernels, are also produced during processing. Among these, hulls and leaves are generated in the largest quantities; however, they are often discarded without further use, contributing to environmental challenges [6, 7]. These residues are sometimes incorporated into soil as organic matter or used in limited quantities as animal feed additives. Nonetheless, due to their high levels of phenolic compounds and tannins, improper disposal or uncontrolled use can negatively affect both animal health and the environment [5]. The main environmental impacts include direct effects such as soil, water, and air pollution, as well as indirect contributions to greenhouse gas emissions [6]. In addition, pistachio fruits must be processed within 24 h after harvest to prevent hull staining caused by trapped moisture [8]. The high‐water content of the hull (up to 70%) also increases the risk of microbial contamination and fermentation, making drying a necessary step in postharvest handling.

In line with the principles of sustainable agriculture and circular economy, the valorization of these by‐products for their bioactive components has become increasingly important [9]. Phytochemicals obtained from agricultural and industrial residues can serve as natural antioxidants and functional food ingredients, offering sustainable alternatives to synthetic compounds [5]. Pistachio by‐products such as hulls and leaves have been reported to be rich in proteins, lipids, phenolics, flavonoids, terpenoids, tannins, minerals, and vitamins. However, information on their biological properties, health‐promoting effects, and potential functional uses remains limited [4, 7, 10]. These compounds are known to exhibit various biological activities, including antioxidant, antimicrobial, anticancer, antidiabetic, and anti‐inflammatory effects. Polyphenols, flavonoids, and tannins in particular, have demonstrated strong antioxidant and pesticidal properties and are considered promising agents in sustainable agriculture due to their roles in plant‐pathogen interactions, such as nematode management [6, 11].

In this study, the hulls and leaves of *P. vera*, which are generally regarded as waste, were selected for comparative evaluation. Phenolic and flavonoid contents, antioxidant and antimicrobial activities, and fatty acid composition were analyzed monthly across these plant organs. Reported values for phenolic content, antioxidant activity, and fatty acid composition of *P. vera* by‐products also vary considerably across studies, reflecting differences in cultivar, growing region and climate, extraction solvent and technique, and analytical method; this variability is part of the motivation for characterizing a single, well‐defined cultivar and orchard across a full growing season. Although the phytochemical composition and biological activities of various pistachio tissues have been examined in previous studies, how these properties change across different phenological stages has received comparatively little attention. This lack of monthly data makes it difficult to identify the periods of highest bioactivity and to standardize collection practices for potential future application. While phenolic content, antioxidant activity, and antimicrobial activity of pistachio hulls and leaves have each been reported individually, few if any studies have combined monthly sampling, two plant organs (leaf and hull), and phenolic, fatty‐acid, antioxidant, and antimicrobial data within a single dataset; this combined monthly picture is the main contribution of this work. To address this, the present study aimed to characterize monthly variation in the biochemical properties and biological activities of *P. vera* leaves and fruit hulls by collecting samples monthly from May, when leaves first emerge, to November, when senescence begins. Analyses were conducted based on methanolic extractions within the framework of standard protocols; total phenolic and flavonoid contents, antioxidant capacities (2,2‐diphenyl‐1‐picrylhydrazyl radical scavenging assay, DPPH; and ferric reducing antioxidant power assay, FRAP), antimicrobial activities, and fatty acid composition (GC‐FID) were determined. In this way, the study aims to establish when these by‐products are most suitable for collection and to characterize their potential for future development in food, pharmaceutical, and cosmetic applications, pending further toxicological and formulation studies.

## 2 Results and Discussion

### 2.1 Total Phenolic and Flavonoid Content

Monthly variation in total phenolic content (expressed as mg gallic acid equivalent per gram dry weight, mg GAE/g DW) and flavonoid content (expressed as µg quercetin equivalent per gram dry weight, µg QE/g DW) was assessed in pistachio leaf and hull tissues (Figure 1). During the studied period, temporal fluctuations were observed in both hull and leaf tissues. Hull tissue showed the highest phenolic content in May, followed by a steady decline through summer and a secondary rise in September. This pattern may be associated with active phenolic biosynthesis during early fruit development, a period when the hull undergoes rapid cell division and might require strong antioxidant protection. Leaf phenolic content, by contrast, remained relatively stable throughout the studied months, potentially reflecting the more constant metabolic demands of photosynthetically active tissue. Descriptive comparisons of hull and leaf phenolic levels from May to September suggest that the two organs follow distinct accumulation patterns during the growing period.

**FIGURE 1:**
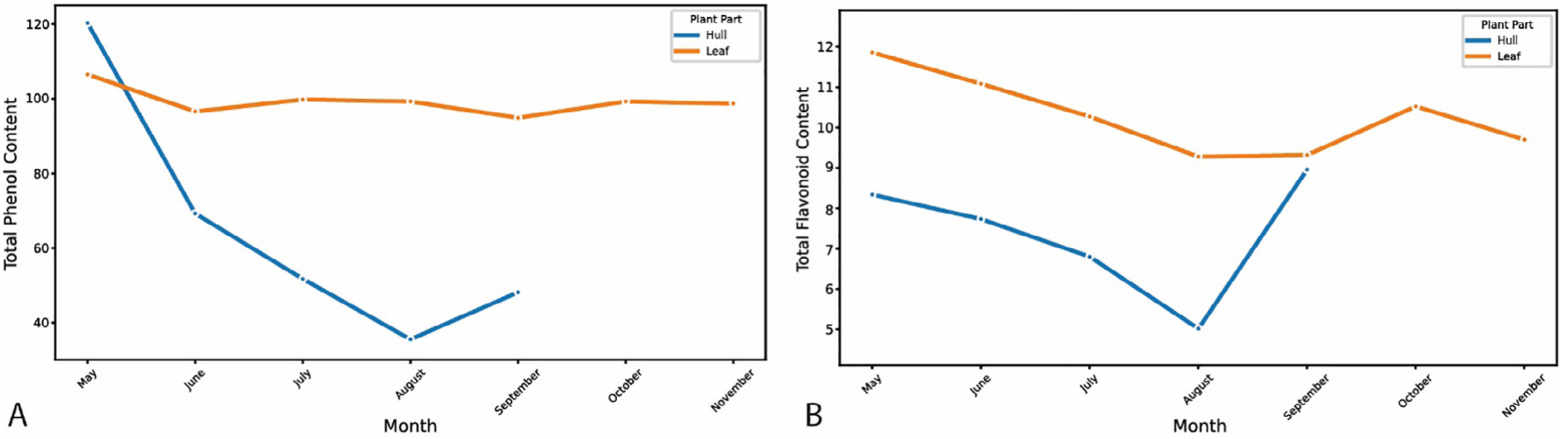
Monthly variation in total phenolic (mg GAE/g DW) (A) and flavonoid (µg QE/g DW) (B) contents of *P. vera* leaf and hull extracts. Data points represent means of analytical triplicates from a single pooled composite sample per month (one composite per tissue) and do not represent independent biological replicates.

Flavonoid content followed a similar temporal pattern. Fluctuations were observed in both hull and leaf tissues across the sampled months. Hull tissues showed the highest flavonoid levels in May, declining through summer to a minimum in August before rising again in September, while leaf flavonoid content remained relatively stable with a slight upward trend toward autumn. Overall, observable differences were noted between the two tissues from May to September. The higher flavonoid accumulation in the hull during early stages is hypothesized to reflect a potential role of these compounds in UV protection and defense against biotic stress during fruit set, a period when the developing fruit is particularly vulnerable.

#### 2.1.1 Antioxidant Activity

DPPH (2,2‐diphenyl‐1‐picrylhydrazyl) radical scavenging activity, expressed as IC_50_ values, showed observable monthly variation in both hull and leaf tissues (Table 1). Lower IC_50_ values indicate stronger radical scavenging capacity. In hull tissue, IC_50_ values increased from May (0.202 mg/mL) to August (0.590 mg/mL), indicating a progressive decrease in antioxidant activity as the fruit matured, before partially recovering in September (0.471 mg/mL). Leaf IC_50_ values, by contrast, remained relatively stable throughout the studied months. Descriptive comparisons of hull and leaf DPPH values from May to September showed observable differences, with the hull consistently showing stronger radical scavenging activity than the leaf during this period. This may be related to the higher phenolic content of the hull in early months, as phenolic compounds are primary contributors to DPPH radical scavenging. Because individual phenolic compounds were not profiled in this study, these antioxidant trends can only be linked to total phenolic and flavonoid content rather than to specific compounds; compound‐resolved profiling (e.g., HPLC‐DAD or LC–MS) is identified as a priority for follow‐up work.

**TABLE 1:** Monthly variation in antioxidant activity of pistachio leaf and hull extracts measured by DPPH radical scavenging and FRAP assays.

	Leaves		Hull	
	DPPH IC_50_ (mg/mL)	FRAP (µmol AA/g DW)	DPPH IC_50_ (mg/mL)	FRAP (µmol AA/g DW)
May	0.125 ± 0.062	20.47 ± 0.30	0.202 ± 0.042	73.78 ± 0.12
June	0.120 ± 0.015	20.87 ± 0.55	0.312 ± 0.029	44.71 ± 2.39
July	0.142 ± 0.002	21.49 ± 0.52	0.529 ± 0.015	30.48 ± 1.01
August	0.132 ± 0.043	23.33 ± 0.11	0.590 ± 0.029	26.47 ± 0.66
September	0.120 ± 0.023	21.92 ± 1.01	0.471 ± 0.050	46.16 ± 1.00
October	0.138 ± 0.003	23.42 ± 0.53	—	—
November	0.152 ± 0.025	23.39 ± 0.50	—	—

Values are expressed as mean ± standard error (SE) of analytical triplicates and represent the analytical repeatability of the pooled composite sample, not independent biological replication.

Ferric reducing antioxidant power (FRAP) values also fluctuated over time in both hull and leaf tissues. Hull tissue showed the highest reducing power in May, which declined through summer before recovering in September. Leaf FRAP values remained relatively steady, with a slight increase toward autumn. Overall comparisons indicated an observable difference between tissues from May to September, with the hull showing greater reducing power than the leaf in the early months. Phenolic content and antioxidant activity followed similar temporal trends in both assays, suggesting that phenolic compounds may be closely linked to antioxidant capacity in these tissues, consistent with findings reported for other *Pistacia* species [12, 13].

The antioxidant and phenolic properties of *P. vera* leaves and hulls have been documented in several studies, though direct monthly comparisons remain scarce. Gök et al. reported that *P. vera* leaf extracts inhibited α‐amylase, α‐glucosidase, and pancreatic lipase, with DPPH IC_50_ values between 7 and 12 µg/mL [12]. Duysak and Özorman recorded a total phenolic content of approximately 210 mg GAE/g and high FRAP activity in methanolic leaf extracts [13]. Alkhathami et al. showed that *P. vera* leaf extract exhibited antioxidant, anti‐inflammatory, and immunomodulatory effects in a rat mammary tumor model [14], while Elakremi et al. demonstrated that incorporation of female tree leaf extracts into bread formulations significantly improved the antioxidant capacity of the final product [15]. These results support the view that *P. vera* leaves are a consistent source of biologically active phenolic compounds across different preparation methods and conditions.

Hull extracts have similarly been shown to contain high concentrations of bioactive constituents. Özbek et al. reported total phenolic contents of 21–39 mg GAE/g and a DPPH IC_50_ of 0.7 mg/mL in microwave‐assisted hull extracts [5]. Şeker and Akbaş reported gallic acid (1.9 and 1.5 mg/g dw) and quercetin (0.025 and 0.009 mg/g dw) as the main phenolic compounds in ethanolic and aqueous hull extracts, with total phenolic contents of 23.3 and 14.7 mg GAE/g dw and antioxidant activity (SC_50_) values of 0.63 and 0.56 mg/mL, respectively [16]. These bioactivities have been linked to the presence of oleic and linoleic acids, which are known to support antioxidant enzyme activity under oxidative stress [16, 17]. Arjeh et al. and Hassan et al. further highlighted that pistachio hulls are rich in phenolic compounds, flavonoids, and tocotrienols with anti‐inflammatory and antimutagenic properties [1, 18].

Species‐level comparisons within the *Pistacia* genus reveal meaningful chemical variation. Harrat et al. found α‐linolenic acid contents of 20%–49% in *Pistacia lentiscus* (*P. lentiscus*) leaves [19], while the present study recorded simultaneously high levels of both linoleic and α‐linolenic acids in *P. vera*, pointing to species‐specific differences in fatty acid metabolism. Khadivi et al. reported total phenolic contents of 99.9–184.3 mg GAE/g DW and antioxidant capacities of 39.1%–82.9% across Iranian *P. vera* cultivars, with a positive correlation between mineral nutrition and phenolic accumulation [20]. These cultivar‐level differences underscore the influence of both genetic background and growing conditions on secondary metabolite profiles. In the present study, leaf total phenolic content ranged from 90.9 to 106.6 mg GAE/g DW across the sampled months, lower than the ∼210 mg GAE/g reported by Duysak and Özorman for methanolic leaf extracts [13] but broadly comparable to the 99.9–184.3 mg GAE/g DW range reported across Iranian *P. vera* cultivars [20]. Hull total phenolic content ranged more widely, from 38.0 to 120.3 mg GAE/g DW, exceeding the 21–39 mg GAE/g reported by Özbek et al. for microwave‐assisted hull extracts [5] and the 14.7–23.3 mg GAE/g dw reported by Şeker and Akbaş [16], although our lowest monthly value (38.0, August) approached the upper end of the Özbek et al. range. These differences most likely reflect cultivar (Barak yıldızı), extraction solvent (methanolic maceration versus microwave‐assisted or aqueous/ethanolic extraction), and the growth stage at which samples were collected, rather than a fundamental difference in phenolic capacity.

Piñeiro et al. identified 26 compounds in a *P. vera* waste decoction, including gallic acid, quercetin, kaempferol, and anacardic acid derivatives, with a total phenolic content of 212.65 mg GAE/g DW and a FRAP value of 7.52 mM TE/g DW [6]. The extract also showed cholinesterase‐inhibitory and nematocidal activities (LC_50_ = 0.12% at 24 h), confirming that *P. vera* processing residues contain a chemically diverse array of bioactive molecules. The present findings are consistent with this body of evidence and further indicate that harvest timing across the sampled months is associated with the bioactive potential of both leaf and hull tissues. This monthly variation may be associated with changes in plant metabolism, particularly shifts in secondary metabolite biosynthesis during active growth and stress adaptation.

### 2.2 Crude Oil and Fatty Acid Contents

Crude oil content increased progressively in both tissues throughout the sampling period (Table 2). In leaves, values rose steadily from 1.72% in May to a peak of 3.96% in October, followed by a slight decline in November. Hull oil content followed a similar pattern, increasing from 4.04% in May to 8.28% in September, the final harvest month, after which no hull samples were available. These values are consistent with previously reported data for *P. vera* hull oil. Barut et al. reported crude oil contents of 8.66% in Kırmızı and Boz varieties and 5.63% in Siirt pistachios [21]. The hull values obtained in the present study are close to those of Kırmızı and Boz varieties and higher than those recorded for Siirt pistachios, which may reflect cultivar‐dependent differences in lipid accumulation capacity.

**TABLE 2:** Monthly variation in crude oil content (%, w/w dry weight) of *P. vera* leaves and hulls.

Collection date	Leaves	Hull
01 May	1.72	4.04
01 June	1.83	6.41
01 July	2.28	7.13
01 August	2.93	8.21
01 September	3.23	8.28
01 October	3.96	—^a^
01 November	3.79	—^a^

^a^No hull sample was available after September (fruit harvest completed).

The fatty acid profiles of both tissues varied across the sampled months and differed between organs (Tables 3 and 4). The unidentified peaks in Table 3 (leaf, July and August) and Table 4 (hull, September) matched the retention time of a DHA methyl ester standard in the Supelco 37 Component FAME Mix used for identification; however, DHA (C22:6 n‐3) is a marine‐derived fatty acid that terrestrial plants lack the enzymatic pathway to synthesize, making this assignment biologically implausible. Without confirmatory mass‐spectrometric identification, these peaks are therefore reported as unidentified components pending further verification. In leaf samples, polyunsaturated fatty acids (PUFAs) were the most abundant group throughout the sampled months, ranging from 40.67% in November to 63.15% in May. α‐Linolenic acid (C18:3) and linoleic acid (C18:2) were the principal PUFAs; ALA peaked in May (37.54%) and again in October (38.28%), while linoleic acid remained relatively stable. Saturated fatty acid (SFA) content ranged from 23.76% in May to 34.58% in September, with palmitic acid (C16:0) as the predominant component across all months. Stearic acid (C18:0) and trace amounts of behenic and tricosanoic acids were detected in specific months. Monounsaturated fatty acid (MUFA) content varied between 13.06% in May and 32.64% in July, driven primarily by a sharp increase in oleic acid (C18:1), which reached 30.58% in July before returning to lower levels. Minor MUFAs, including cis‐10‐heptadecanoic acid and nervonic acid, were detected in selected months.

**TABLE 3:** Monthly variation (%) of fatty acid composition in *P. vera* leaves.

Carbon number	Fatty acids	May	June	July	August	September	October	November
C14:0	Myristic acid	—	—	1.62 ± 0.05	5.82 ± 0.17	6.49 ± 0.19	7.39 ± 0.22	6.02 ± 0.17
C16:0	Palmitic acid	19.88 ± 0.57	22.09 ± 0.62	19.45 ± 0.56	21.0 ± 0.61	24.09 ± 0.71	17.35 ± 0.51	16.34 ± 0.47
C18:0	Stearic acid	—	1.82 ± 0.05	2.91 ± 0.09	2.27 ± 0.07	4.0 ± 0.12	2.90 ± 0.09	2.54 ± 0.08
C22:0	Behenic acid	3.88 ± 0.12	1.91 ± 0.05	0.77 ± 0.02	1.06 ± 0.03	—	—	0.44 ± 0.01
C23:0	Tricosanoic acid	—	2.63 ± 0.06	0.48 ± 0.01	1.75 ± 0.05	—	—	1.13 ± 0.03
C24:0	Lignoceric acid	—	—	0.78 ± 0.02	1.48 ± 0.04	—	—	1.19 ± 0.06
**Total SFAs**		**23.76** ± 0.71	**28.45** ± 0.84	**26.01** ± 0.78	**33.38** ± 1.01	**34.58** ± 1.02	**27.64** ± 0.85	**27.66** ± 0.83
C16:1	Palmitoleic acid	—	—	0.80 ± 0.02	—	—	—	—
C17:1	Cis‐10‐heptadecanoic acid	2.13 ± 0.06	2.12 ± 0.06	0.84 ± 0.03	3.28 ± 0.11	3.53 ± 0.10	2.07 ± 0.06	1.47 ± 0.04
C18:1	Oleic acid	10.93 ± 0.32	14.84 ± 0.44	30.58 ± 0.91	14.42 ± 0.43	14.65 ± 0.44	18.60 ± 0.55	30.0 ± 0.89
C24:1	Nervonic acid	—	—	0.42 ± 0.01	—	—	—	—
**Total MUFAs**		**13.06** ± 0.39	**16.96** ± 0.51	**32.64** ± 0.97	**17.70** ± 0.40	**18.18** ± 0.54	**20.67** ± 0.62	**31.47** ± 0.94
C18:2	Linoleic acid	25.62 ± 0.77	19.14 ± 0.56	27.75 ± 0.81	15.60 ± 0.47	15.66 ± 0.46	13.37 ± 0.41	18.82 ± 0.56
C18:3	α‐Linolenic acid	37.53 ± 1.13	35.43 ± 1.06	12.85 ± 0.39	31.82 ± 0.95	31.61 ± 0.94	38.28 ± 1.14	21.12 ± 0.64
C20:3	Cis‐8,11,14‐eicosatrienoic acid	—	—	—	—	—	—	0.73 ± 0.02
—	Unidentified component (RT ≈ 42.1 min)^a^	—	—	0.69 ± 0.02	1.13 ± 0.04	—	—	—
**Total PUFAs (including unidentified component)**		**63.15** ± 1.89	**54.57** ± 1.65	**41.29** ± 1.24	**48.55** ± 1.46	**47.27** ± 1.42	**51.65** ± 1.55	**40.67** ± 1.22

Values are expressed as mean ± standard error (SE) of analytical triplicates and represent the analytical repeatability of the pooled composite sample, not independent biological replication.

^a^Retention time (RT ≈ 42.1 min) matches the certified DHA methyl ester standard in the Supelco 37 FAME Mix, but DHA is biologically implausible in terrestrial plant tissue; identification is pending confirmatory analysis, currently in progress.

**TABLE 4:** Monthly variation (%) of fatty acid composition in *P. vera* hulls.

Carbon number	Fatty acids	May	June	July	August	September
C16:0	Palmitic acid	22.43 ± 0.67	22.73 ± 0.68	21.52 ± 0.65	22.71 ± 0.68	21.04 ± 0.63
C18:0	Stearic acid	2.29 ± 0.07	2.23 ± 0.07	2.41 ± 0.07	2.82 ± 0.08	—
C22:0	Behenic acid	—	—	0.27 ± 0.01	—	—
C24:0	Lignoceric acid	—	—	0.49 ± 0.01	—	—
**Total SFAs**		**24.72 ± 0.74**	**24.96 ± 0.75**	**24.69 ± 0.74**	**25.53 ± 0.77**	**21.04 ± 0.63**
C16:1	Palmitoleic acid	—	—	0.51 ± 0.02	—	—
C17:1	Cis‐10‐heptadecanoic acid	0.56 ± 0.02	2.10 ± 0.06	0.45 ± 0.01	2.25 ± 0.07	—
C18:1	Oleic acid	11.97 ± 0.36	21.15 ± 0.66	21.56 ± 0.65	27.14 ± 0.81	20.90 ± 0.63
**Total MUFAs**		**12.53 ± 0.38**	**23.25 ± 0.67**	**22.52 ± 0.68**	**29.39 ± 0.88**	**20.90 ± 0.63**
C18:2	Linoleic acid	39.53 ± 1.19	35.65 ± 1.07	49.65 ± 1.49	32.42 ± 0.97	23.98 ± 0.72
C18:3	α‐Linolenic acid	23.20 ± 0.70	16.13 ± 0.48	3.11 ± 0.09	12.64 ± 0.38	14.03 ± 0.42
—	Unidentified component (RT ≈ 41.4 min)^a^	—	—	—	—	20.04 ± 0.60
**Total PUFAs (including unidentified component)**		**62.73 ± 1.76**	**51.78 ± 1.67**	**52.76 ± 1.58**	**45.06 ± 1.35**	**58.05 ± 1.74**

Values are expressed as mean ± standard error (SE) of analytical triplicates and represent the analytical repeatability of the pooled composite sample, not independent biological replication.

^a^Retention time (RT ≈ 41.4 min) matches the certified DHA methyl ester standard in the Supelco 37 FAME Mix, and is distinct from nervonic acid (RT ≈ 40.1 min), but DHA is biologically implausible in terrestrial plant tissue; identification is pending confirmatory analysis, currently in progress.

In hull samples, SFA content was more stable than in leaves, varying between 21.04% in September and 25.53% in August, with palmitic acid as the predominant component across all months. MUFA content ranged from 12.53% in May to 29.39% in August, with oleic acid rising from 11.97% in May to 27.14% in August as the fruit developed. PUFAs were the most abundant fatty acid group throughout the sampled months, ranging from 45.06% in August to 62.73% in May (September, at 58.05%, was also elevated by the unidentified component discussed above). Linoleic acid peaked at 49.65% in July, while ALA dropped from 23.20% in May to 3.11% in July before partially recovering in later months.

The fatty acid profiles of *P. vera* leaves and hulls differ considerably from those of the kernel, and published data on these tissues remain scarce. Aslan and Orhan reported that *P. vera* leaves are rich in α‐linolenic acid [22], consistent with the present findings. However, the palmitic acid and total SFA levels recorded here were notably higher than those reported by the same authors, which may reflect differences in cultivar, growing conditions, or sampling period.

Previous studies on pistachio hull oil have consistently reported oleic acid as the dominant fatty acid (42%–48%), followed by linoleic acid (20%–27%), α‐linolenic acid (4.9‐9.0%), and palmitic acid (12%–15%) [5, 23, 24, 25]. The present study, however, recorded lower oleic acid proportions and higher levels of palmitic, linoleic, and α‐linolenic acids than these reported ranges. These differences are likely related to the characteristics of the Barak yıldızı variety, which has not been previously characterized for hull fatty acid composition. Environmental conditions, harvest timing, and extraction methodology may also have contributed to the observed variation. The predominance of PUFAs in both tissues, particularly α‐linolenic acid in leaves, is consistent with their structural role in maintaining membrane fluidity and supporting photosynthetic processes in metabolically active tissues [26]. The more pronounced monthly fluctuations observed in hull linoleic acid levels, likely reflect lipid remodeling processes occurring during fruit maturation [27].

Kernel oil is characterized by a high oleic acid content, generally ranging from 56% to 72% [20, 28, 29], which is substantially higher than the MUFA levels recorded in either leaves or hulls in the present study. Palmitic acid content in leaves and hulls (16%–24%) was approximately twice that reported for kernel oil. This compositional difference reflects the distinct physiological functions of these tissues: oleic acid‐rich kernel oil serves energy storage and oxidative stability, while the higher PUFA content of leaves and hulls is consistent with their roles in membrane integrity and photosynthetic activity. In hull tissue, oleic acid was predominantly negatively associated with the antioxidant parameters, whereas linoleic acid was positively associated, indicating that the contribution of individual fatty acids to oxidative defense during fruit ripening is tissue‐dependent. Boukelouaa et al. reported that *P. lentiscus* fruits contained 51.18% oleic acid and 21.86% linoleic acid [30], a MUFA‐dominant profile that contrasts with the PUFA‐rich composition recorded here for *P. vera* leaves and hulls, pointing to meaningful interspecific variation in lipid metabolism within the *Pistacia* genus. The higher SFA content observed in leaves and hulls compared to the kernel may also relate to their direct exposure to environmental stressors; saturated fatty acids contribute to membrane rigidity and protection against temperature fluctuations and oxidative stress [31].

The fatty acid data show that *P. vera* leaves and hulls are rich in PUFAs, particularly linoleic and α‐linolenic acids, and differ substantially from the kernel in their lipid composition. The higher SFA levels compared to previously reported values for similar tissues likely reflect the characteristics of the Barak yıldızı variety and the environmental conditions at the sampling site. The monthly and tissue‐specific variation in fatty acid profiles observed here, when considered alongside the phenolic and antioxidant data, points to a coordinated biochemical response to developmental and environmental cues across the growing season [32]. Future work accounting for cultivar, climate, and extraction methodology will help clarify the sources of this variability and better define the nutritional and industrial value of these by‐products.

### 2.3 Association of Specific Fatty Acids With Antioxidant Activity

An exploratory Pearson correlation analysis was conducted to observe preliminary covariation between fatty acid composition and antioxidant capacity in leaf and hull tissues across the five monthly sampling points (*n* = 5). The fatty acids included in the analysis (palmitic acid (C16:0), stearic acid (C18:0), oleic acid (C18:1), linoleic acid (C18:2), and α‐linolenic acid (C18:3)) were selected because they were consistently detected across all sampling months (May–September) and represent the predominant saturated, monounsaturated, and PUFAs in both tissues. The descriptive heatmap analysis revealed different covariation patterns in the two tissues (Figure 2). In leaf tissue (A), oleic acid (C18:1) and linoleic acid (C18:2) showed negative correlations with α‐linolenic acid (*r* = −0.98 and *r* = −0.89, respectively). Oleic acid (C18:1) displayed a weak negative association with DPPH and FRAP (*r* = −0.42 and *r* = −0.37, respectively). Palmitic acid (C16:0) exhibited positive correlations with FRAP (*r* = 0.74), DPPH (*r* = 0.45), and flavonoid content (*r* = 0.60), while stearic acid (C18:0) showed a positive correlation with flavonoid content (*r* = 0.81). In hull tissue (B), oleic acid (C18:1) was strongly and negatively correlated with FRAP (*r* = −0.95), DPPH (*r* = −0.91), flavonoid content (*r* = −0.90), and phenolic content (*r* = −0.99). α‐Linolenic acid showed strong positive correlations with FRAP (*r* = 0.83), DPPH (*r* = 0.88), and phenolic content (*r* = 0.73). Phenolic and flavonoid contents were positively associated with both antioxidant assays, which aligns with the well‐established role of phenolics as primary antioxidants in plant extracts.

**FIGURE 2:**
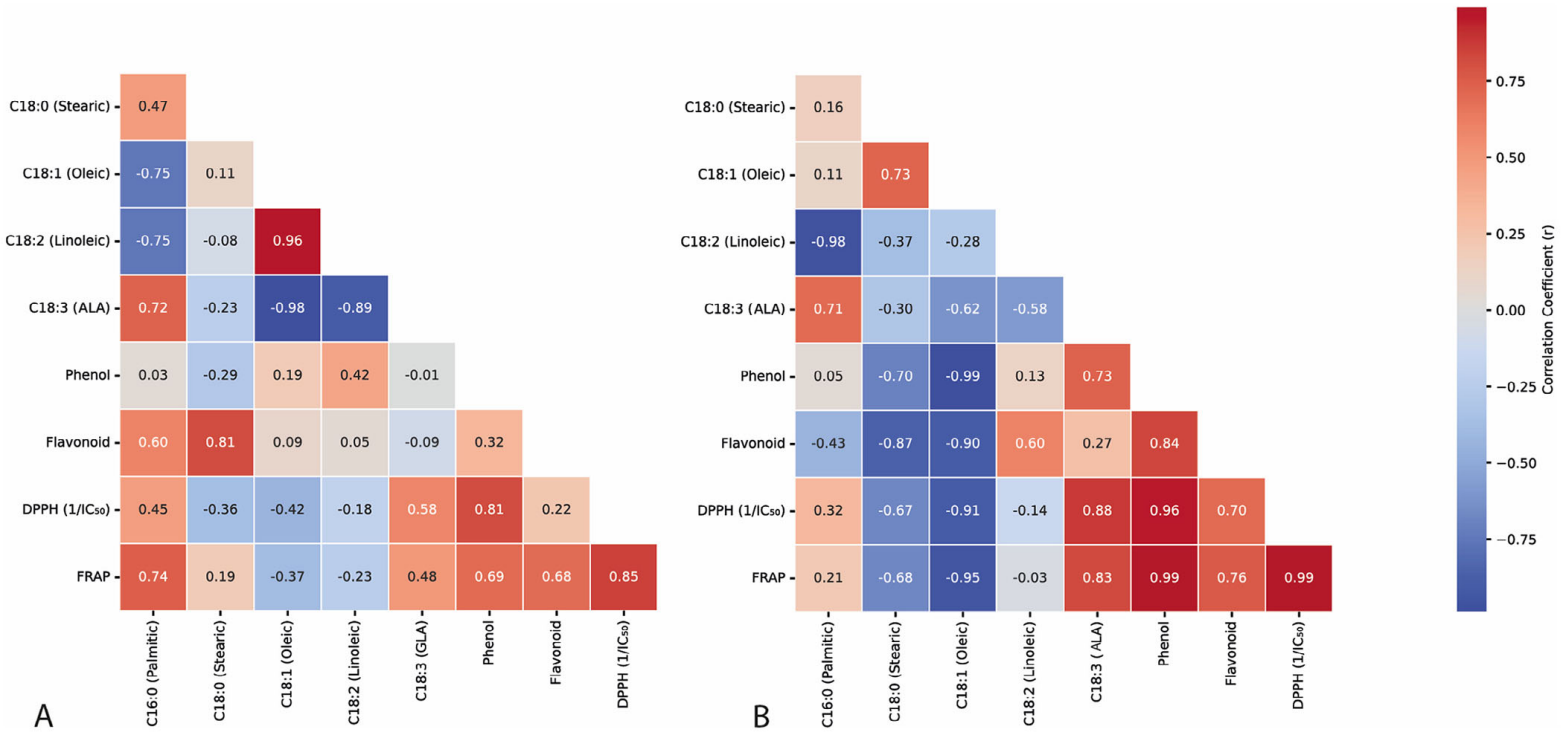
Exploratory heatmap visualization of Pearson correlation coefficients (*r*) between selected fatty acids, total phenolic and flavonoid contents, and antioxidant activity (FRAP and DPPH) in *Pistacia vera* (A) leaf and (B) hull tissues. Data represent preliminary covariation across five monthly time points (*n* = 5). Due to the small sample size, these correlations are purely descriptive and do not imply causal or mechanistic relationships.

In hull tissue, oleic acid (C18:1) showed strong negative correlations with both FRAP (*r* = −0.95) and DPPH (1/IC_50_) (*r* = −0.91), as well as with phenolic (*r* = −0.99) and flavonoid (*r* = −0.90) contents. This pattern may reflect an inverse temporal association between lipid and phenolic metabolism during fruit maturation: as oleic acid accumulated toward harvest, phenolic compound levels (the primary contributors to DPPH and FRAP activity) declined. However, with only *n* = 5 monthly observations this association should be interpreted descriptively rather than causally, and further studies are needed to confirm these preliminary trends. A similar pattern has been reported in olive fruit, where phenolic content decreased as the fruit matured while fatty acid composition shifted toward higher oleic acid proportions [33]. Linoleic acid (C18:2) also showed a positive correlation with antioxidant parameters, particularly with DPPH (*r* = 0.80). Palmitic acid (C16:0), by contrast, showed weak or inverse relationships with antioxidant capacity; while this is often observed for saturated fatty acids, these limited observations cannot establish mechanistic roles.

Both tissues were rich in PUFAs, particularly linoleic and α‐linolenic acids, though their profiles changed differently across the sampled months. Leaf fatty acid composition was also variable, while hull samples showed more marked fluctuations, especially during the fruiting period (July–August). The correlation data indicate that individual fatty acids play distinct roles in antioxidant activity depending on the tissue. In leaf tissue, the positive associations between palmitic acid and antioxidant parameters likely reflect its co‐occurrence with phenolic compounds during active growth, rather than direct radical scavenging activity. In hull tissue, the monthly decline in phenolic content co‐occurred with oleic acid accumulation and reduced antioxidant capacity [33]; given the limited number of monthly observations, phenolic content remains the more likely explanation, though this cannot be confirmed from this dataset. These results are consistent with findings in other plant species, where phenolic compounds have been identified as the dominant contributors to DPPH and FRAP activity across seasons [34, 35].

### 2.4 Antimicrobial Activity of Leaf and Hull Extracts

Leaf extracts showed moderate antimicrobial activity that varied across the sampled months (Figure 3). The highest inhibition zones were recorded against *Serratia marcescens* (*S. marcescens*) and *Micrococcus luteus* (*M. luteus*) ATCC 9341, reaching 28‐31 mm in certain months. Against *Staphylococcus aureus* (*S. aureus*) strains and the yeasts *Candida albicans* (*C. albicans*) and *Saccharomyces cerevisiae* (*S. cerevisiae*), inhibition zones generally remained below 20 mm. Hull extracts showed a somewhat narrower but more consistent activity profile throughout the sampled months; *S. marcescens* and *M. luteus* were again the most susceptible strains, while *C. albicans* and *S. cerevisiae* were the least affected (zones ≈ 10–15 mm). Both extracts produced inhibition zones against the bacterial strains; these zones are not directly comparable to those of the ampicillin positive control, because complex plant extracts and small antibiotic molecules differ in their diffusion through agar, while nystatin confirmed selective antifungal efficacy against *C. albicans* and *S. cerevisiae* only. Leaf extracts showed stronger antibacterial effects overall, while hull extracts maintained more stable but slightly lower activity. The observed monthly differences suggest that secondary metabolite concentrations fluctuate across the sampled months, with harvest timing having a clear influence on antimicrobial efficacy.

**FIGURE 3:**
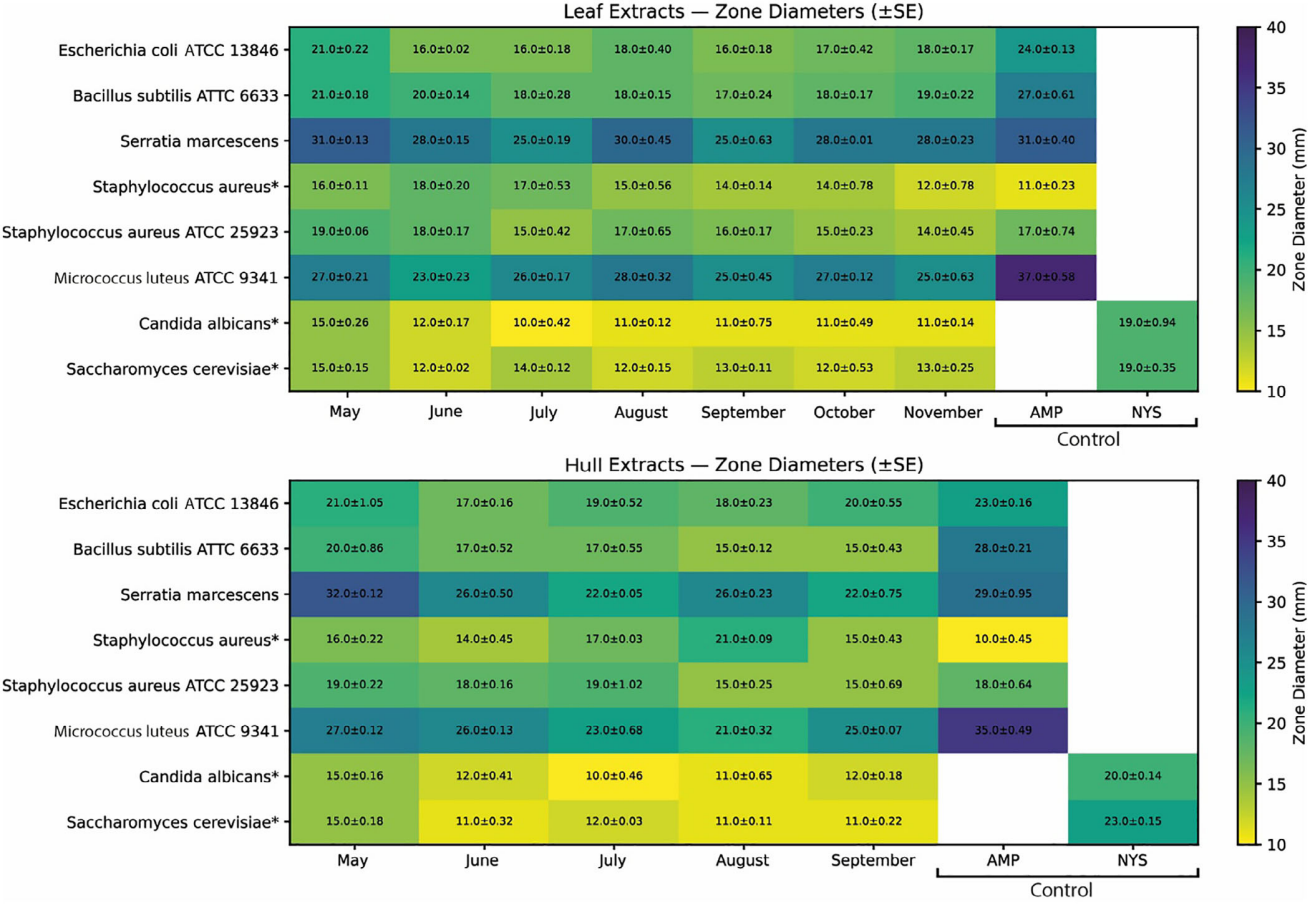
The heatmaps illustrate the variation in antimicrobial activity of the leaf and hull extracts across different sampling months against a range of bacterial and fungal test microorganisms. The color intensity represents the zone of inhibition (mm), with darker shades indicating higher antimicrobial activity. Both datasets are scaled between 10 and 40 mm to ensure direct visual comparability. Data represent means ± SE of analytical replicates from a single pooled composite sample per month.

The minimum inhibitory concentration (MIC) values of *P. vera* leaf and hull extracts against selected bacterial and fungal strains are presented in Table 5. Both extracts showed antimicrobial activity within a concentration range of 8–32 mg/mL, with differences depending on the microorganism and sampling month. Leaf extracts generally required lower concentrations to inhibit microbial growth than hull extracts. From June to August, MIC values of leaf extracts fell to their lowest levels (8‐16 mg/mL) against *Escherichia coli*, *S. aureus* ATCC 25923, *S. marcescens*, *M. luteus*, and *S. cerevisiae*. Hull extracts, by contrast, typically required concentrations of 16–32 mg/mL and showed less variation across months. For *S. marcescens*, *S. aureus* ATCC 25923, and *M. luteus* ATCC 9341, hull MIC values remained at 32 mg/mL throughout the sampled months. The stronger inhibitory effect of leaf extracts is consistent with their higher phenolic and flavonoid contents compared to hull extracts. The observed MIC range (8–32 mg/mL) indicates moderate, rather than strong, antimicrobial potency compared with purified antibiotics; using them industrially or in food preservation would first require further purification, toxicity testing, stability testing, and testing in real food products, none of which were done here.

**TABLE 5:** MIC values of *P. vera* leaf and hull extracts.

	May		June		July		August		September		October		November	
Microorganisms	Leaf	Hull	Leaf	Hull	Leaf	Hull	Leaf	Hull	Leaf	Hull	Leaf	Hull	Leaf	Hull
*E. coli *ATCC 13846	32	32	8	32	8	32	8	16	8	16	8	—	8	—
*B. subtilis ATCC 6633*	32	32	16	32	16	16	32	16	16	32	16	—	16	—
*S. marcescens*	32	32	16	32	16	32	16	32	16	32	16	—	16	—
*S. aureus* ^a^	32	32	8	32	16	16	32	8	16	32	16	—	16	—
*S. aureus ATCC25923*	32	32	16	32	16	32	16	32	32	32	16	—	16	—
*M. luteus ATCC9341*	32	32	32	32	16	32	16	32	16	32	16	—	16	—
*C. albicans* ^a^	16	32	16	32	16	16	32	32	16	32	16	—	16	—
*S. cerevisiae* ^a^	32	32	16	32	16	16	16	16	16	16	16	—	16	—

Abbreviation:—, Not tested)

^a^Clinical isolate

Several studies have examined the antimicrobial potential of *P. vera* using different plant parts, extraction methods, and solvents. Şeker and Akbaş reported MIC values of 0.8–49.0 mg/mL for ethanolic hull extracts and 9.6–82.5 mg/mL for aqueous hull extracts against *Enterococcus faecalis*, *S. aureus*, *Streptococcus uberis*, *Bacillus cereus* (*B. cereus*), and *Bacillus subtilis* (*B. subtilis*) [16]. The lower MIC value reported in their study (0.8 mg/mL) indicates stronger inhibitory potency than observed in the present study. This difference may be related to the extraction method used, as they employed microwave‐assisted extraction, whereas conventional sonication was used in the present study. The use of ethanol instead of methanol may also have contributed to the difference. Ghalem and Mohamed extracted essential oils from *P. vera* resins and reported inhibition zones of 7–11.5 mm against *E. coli*, *Proteus* spp., and *S. aureus*, with MIC values of 6–12 mg/mL [36]. The inhibition zones were smaller than those observed in the present study, whereas the MIC values were within a similar range. This variation may be associated with differences in the type of extract and plant material used. Şermet investigated *P. vera* shell extracts prepared using different solvents and found that the methanol extract showed the highest antimicrobial activity, with MIC values ranging from 0.5 to 2 mg/mL [37]. These values were lower than those observed in the present study, indicating stronger inhibitory potency. These values were lower than those obtained in the present study, indicating stronger inhibitory activity. The difference may be related to the use of shell rather than hull tissue and to differences in the extraction procedure. İnci and Kırbağ investigated red hull extracts obtained from *P. vera* grown in Gaziantep and reported inhibition zones of up to 20 mm against *Candida glabrata* and zones of 8–13 mm against *B. subtilis* for methanol extracts [38]. The larger inhibition zones observed in the present study, particularly against *S. marcescens* and *M. luteus*, may be related to differences in harvest time and regional variation in the phytochemical composition of *P. vera* extracts.

The stronger antibacterial activity of leaf extracts is consistent with their higher phenolic and flavonoid contents. Phenolic compounds previously reported in *P. vera* by other studies, such as gallic acid and quercetin, are known to disrupt microbial membranes, interfere with energy metabolism, and inhibit nucleic acid synthesis [39, 40]. Terpenoids such as α‐pinene act through hydrophobic interactions with membrane lipids, causing cellular damage [41, 42]. Both mechanisms have been implicated in the inhibitory activity of *P. vera* hull oils and extracts against *B. cereus* and *S. aureus* [43, 44].

Gram‐positive bacteria were generally more susceptible to both extracts than Gram‐negative strains, consistent with earlier reports on *P. vera* hull and resin extracts [37, 38, 44, 45]. This pattern is explained by structural differences between the two groups: Gram‐positive bacteria lack an outer membrane, which allows hydrophobic antimicrobial compounds to penetrate more easily, while the outer membrane of Gram‐negative bacteria acts as an additional permeability barrier. In food system applications, Fattahifar et al. reported reduced bacterial growth in fresh mushrooms treated with pistachio hull extract during cold storage [45], and Rafiee et al. demonstrated that nanoliposome‐encapsulated *P. vera* hull extracts provided antimicrobial protection in mayonnaise at levels comparable to 1000 mg/kg sodium benzoate [46].

These results indicate that the antimicrobial activity of *P. vera* extracts depends on the tissue type, monthly changes in phytochemical composition, and extraction conditions. The comparatively higher inhibition recorded against *S. marcescens* and *M. luteus*, combined with the food system data reported in recent literature, suggests that pistachio leaf and hull extracts could become natural antimicrobial ingredients for food preservation and pharmaceutical use, though this would first require further purification, toxicity testing, and formulation work. It should also be noted that agar‐diffusion zone diameters are influenced by the differential diffusion rates of complex, relatively hydrophobic plant extracts compared with small, more diffusible antibiotic molecules; MIC values, which are less affected by this limitation, are therefore the more reliable comparator between extracts and the ampicillin/nystatin positive controls used here.

## 3 Conclusions

This study showed that *P. vera* leaves and fruit hulls contain substantial amounts of bioactive compounds whose concentrations change across the studied months. Both tissues were rich in PUFAs, with linoleic acid (C18:2, 13%–50%) and α‐linolenic acid (C18:3, 3%–38%) as the principal components, while total saturated fatty acid content ranged between 24%–35% in leaves and 21%–26% in hulls. Total phenolic and flavonoid contents peaked in spring and showed observable monthly fluctuations, with hull antioxidant activity following a similar temporal pattern and peaking in spring, while leaf antioxidant activity remained comparatively stable across the studied period. Exploratory correlation analysis suggested that phenolic compounds tracked antioxidant capacity most closely in both tissues, while the preliminary covariation between fatty acid composition and antioxidant activity was tissue‐dependent. Antimicrobial assays recorded inhibition zones of up to 28–31 mm against *S. marcescens* and *M. luteus*, with leaf extracts showing stronger activity than hull extracts across most months under these experimental conditions.

This study has some limitations. Samples were pooled from three trees into one composite per month, so the reported replicates are analytical rather than biological, and the results come from a single cultivar, orchard, and growing season; consequently, the findings are presented descriptively rather than as statistical inferences. Phenolic and fatty acid identifications were also based on total values and retention‐time matching rather than compound‐specific profiling and confirmatory techniques (e.g., GC–MS). Future work covering more cultivars, locations, and seasons, together with compound‐level analysis, would help confirm and extend these findings, including the preliminary spring harvest recommendation.

The temporal data presented here show that the bioactive potential of *P. vera* leaves and hulls varies over time, and that within the conditions of this single‐season, single‐cultivar study, spring appeared to be a favorable period for harvesting material with high phenolic and antioxidant activity; this observation is preliminary and requires confirmation across additional seasons, cultivars, and locations before it can inform general harvesting recommendations. These findings provide a practical basis for optimizing collection strategies and identify pistachio processing waste as a potential future source of antioxidants and antimicrobial compounds for food, pharmaceutical, and cosmetic applications, pending further toxicological, formulation, and stability studies. Future studies should examine additional cultivars and growing regions to determine how genetic and environmental factors influence seasonal bioactive profiles, and should explore the incorporation of these extracts into food matrices to evaluate their functional performance under real application conditions.

## 4 Experimental Section

### 4.1 Plant Material

The plant materials of *P. vera* used in this study were collected in 2021 from a commercial pistachio orchard in Kıvırcık Village, Nizip district of Gaziantep Province, Türkiye (GPS: 36°49′55″N, 37°56′58″E; altitude: 376 m). The orchard consisted of the Barak yıldızı cultivar, with trees approximately 50 years old and reaching 3–4 m in height. Samples were collected from three marked trees at each sampling date and pooled prior to analysis to obtain a representative composite sample for each time point. The experimental unit at each time point is therefore this pooled composite sample; the triplicate measurements reported throughout reflect analytical replicates of that composite rather than independent biological replicates. Sampling was carried out at monthly intervals from May, marking the onset of vegetative growth, until September, when the final commercial fruit harvest was completed (Figure 4); hull sampling ended with this harvest, whereas leaves remained on the trees and continued to be sampled through November to capture post‐harvest and senescence stages. The timing of sample collection was planned according to the phenological cycle of the plant, including fruit set, maturation, final harvest, and post‐harvest stages.

**FIGURE 4:**
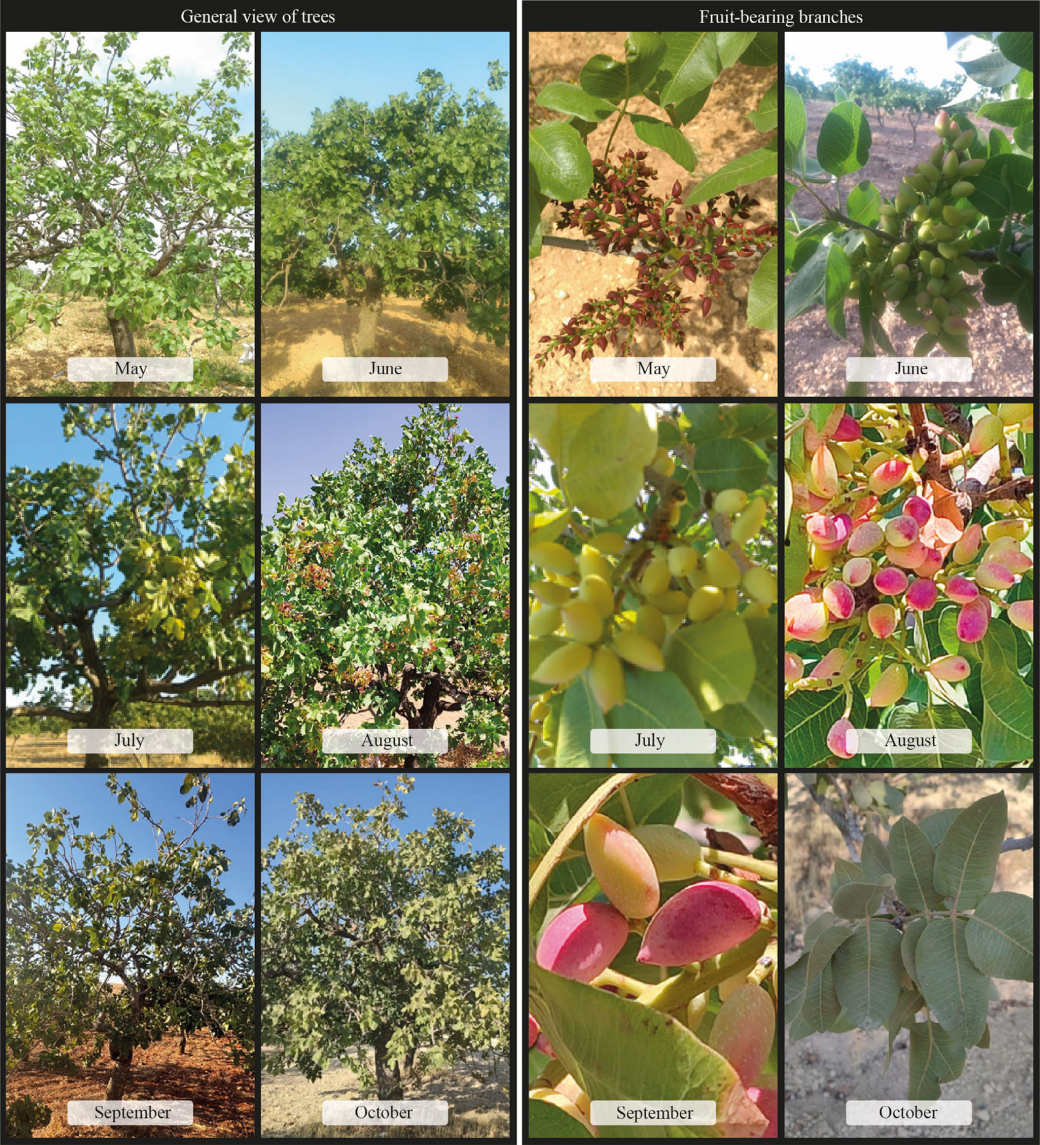
Monthly phenological development of *P. vera* plants from May to October.

### 4.2 Sample Preparation

Collected plant samples were immediately transported to the laboratory. Leaves were washed with tap water to remove surface impurities. The soft outer hulls of the fruits were manually separated, and the inner hard shells were cracked open using a hand cracker to remove the seeds. The cleaned samples were air‐dried at room temperature in a shaded, well‐ventilated environment until a constant weight was achieved. After drying, the materials were separated by organ type, ground to a particle size of 60 mesh (250 µm) using a laboratory mill, and stored in airtight containers protected from moisture and light in a freezer (−20°C) until analysis.

### 4.3 Extraction Procedure

Extraction was performed using a modified version of the method described by Comlekcioglu and Kutlu [47], with the main modification being a three‐cycle sequential extraction procedure. One gram of powdered leaf or hull sample was weighed and 10 mL of methanol was added. The mixture was sonicated for 20 min in an ultrasonic water bath (40 kHz) maintained below 35 ± 2°C, then filtered. The residue was re‐extracted twice under the same conditions, each time with a fresh 10 mL portion of methanol, resulting in a total sonication time of 60 min. The filtrates from all three cycles were combined and concentrated under reduced pressure using a rotary evaporator (water bath 40°C, 300–340 mbar, 80–120 rpm). The concentrated extracts were stored at −20°C until further analysis. Extraction yields (mg dry extract per g dry sample) were 46.04 (May), 45.32 (June), 43.62 (July), 40.77 (August), and 46.16 (September) for hull, and 42.74 (May), 41.58 (June), 40.91 (July), 39.82 (August), 40.21 (September), 40.48 (October), and 39.58 (November) for leaf.

### 4.4 Crude Oil Extraction and Fatty Acid Composition

Crude oil was extracted using a Foss Soxtec 2055 automated fat extraction system with n‐hexane as the solvent, following the procedure of Comlekcioglu et al. [48]. Approximately 3–5 g of ground sample was subjected to automated extraction at 135°C, and crude oil content was calculated gravimetrically after drying and cooling. Each monthly crude‐oil value was obtained from a single gravimetric extraction of the pooled composite sample, so no extraction‐level variability is available. For fatty acid analysis, oil samples were converted to fatty acid methyl esters (FAMEs) prior to GC analysis. Crude‐oil extraction (for gravimetric yield) and the oil sample used for FAME/GC fatty acid analysis were obtained from separate, independent Soxtec extraction runs each month, rather than from a single extraction batch. Briefly, 0.1 g of oil was placed in a 15 mL capped tube, and 1 mL of 2N methanolic KOH solution was added. The mixture was vortexed for 2 min and allowed to stand for 15 min to allow transesterification. Subsequently, 10 mL of n‐hexane was added and the mixture was thoroughly mixed. Phase separation was achieved by centrifugation at 7000 rpm for 10 min, and 0.5 µL of the upper hexane phase was injected into the GC system.

Fatty acid composition was analyzed using a Shimadzu GC‐2025 system equipped with a flame ionization detector (FID) and a Teknokroma TR‐CN100 fused‐silica capillary column (100 m × 0.25 mm i.d. × 0.20 µm film thickness). The oven temperature program was: 80°C (2 min hold), increased at 5°C/min to 140°C (held 2 min), then at 3°C/min to 240°C, held for 5 min; total analysis time was approximately 54 min. The injector and detector temperatures were set to 240°C and 250°C, respectively. Helium was used as the carrier gas in constant‐pressure mode (100 kPa), corresponding to a column flow of 0.33 mL/min and a linear velocity of 8.5 cm/s; the total flow was 50 mL/min and the purge flow 3 mL/min. Makeup gas (helium) was supplied to the detector at 30 mL/min, and the FID was operated with hydrogen at 40 mL/min and dry air at 400 mL/min. The injection volume was 0.5 µL in split mode. Fatty acids were identified by comparison with a Supelco 37 Component FAME Mix certified standard. Identification was based on retention‐time matching to this certified standard only, without confirmatory mass‐spectrometric analysis; minor and trace components should therefore be regarded as tentative identifications.

### 4.5 Determination of Total Phenolic and Flavonoid Content

Total phenolic content was determined using the Folin–Ciocalteu reagent method following the procedure of Blainski et al. [49]. Plant extracts were prepared at 1 mg/mL in distilled water. The extract was mixed with diluted Folin–Ciocalteu reagent (1:3), followed by addition of 10% Na_2_CO_3_ and distilled water. The mixture was incubated in the dark for 30 min and absorbance was measured at 700 nm using a spectrophotometer (Perkin‐Elmer Lambda EZ 150, USA). Results were expressed as total Folin‐reactive compounds (mg gallic acid equivalent per gram of dry weight, mg GAE/g DW) using a gallic acid calibration curve; because pigments and reducing sugars present in *P. vera* extracts can also react with the reagent, these values are interpreted as an index of Folin‐reactive compounds rather than as an absolute measure of phenolic content.

Total flavonoid content was determined spectrophotometrically according to Chang et al. [50]. Plant extract (1 mg/mL) was mixed with methanol, 10% AlCl_3_, and 1 M potassium acetate in distilled water. The mixture was incubated at room temperature for 30 min and absorbance was recorded at 415 nm. Quercetin standard solutions were prepared at concentrations of 25–200 µg/mL. Results were expressed as µg quercetin equivalent per gram of dry weight (µg QE/g DW).

### 4.6 Determination of Antioxidant Activity

#### 4.6.1 DPPH Assay

DPPH radical scavenging activity was determined according to Brand‐Williams et al. [51] with slight modifications. A freshly prepared methanolic DPPH solution (5 mg/100 mL) was used. Ascorbic acid, the reference standard, was tested at eight concentrations from 0.00625 to 0.325 mM prepared from a 1 mM stock. Each extract was tested over a five‐point concentration series in methanol, the range being adjusted for each extract on the basis of preliminary assays (from 0.0156–0.25 mg/mL for the more active extracts up to 0.25–1.5 mg/mL for the less active ones) so that the 50% inhibition point fell within the tested range. For each point, 100 µL of standard or extract was mixed with 950 µL of DPPH solution; a blank (100 µL methanol + 950 µL DPPH) was included in each run. After incubation in the dark at room temperature for 15 min, absorbance was measured at 515 nm. All measurements were carried out in triplicate. IC_50_ values, defined as the extract concentration giving 50% inhibition, were obtained by linear regression of inhibition against concentration (*R* ^2^ = 0.970–0.998) and are expressed for the extracts on a mass basis (mg/mL); the ascorbic acid standard is expressed in mM. Antioxidant activity was calculated as follows:

Antioxidantactivity(%)=[(Acontrol−Asample)/Acontrol]×100

#### 4.6.2 FRAP Assay

Ferric reducing antioxidant power was assessed according to Benzie and Strain [52]. The FRAP reagent was prepared by mixing acetate buffer, TPTZ solution, and ferric chloride solution. Plant extract was added to distilled water, followed by the FRAP reagent, and the mixture was incubated at room temperature for 5 min. Absorbance was measured at 593 nm. Results were expressed as µmol ascorbic acid equivalent per gram of dry weight (µmol AA/g DW) using an ascorbic acid calibration curve. All measurements were carried out in triplicate.

### 4.7 Determination of Antimicrobial Activity

Antimicrobial activity was evaluated by the agar well diffusion method according to Collins et al. [53]. Test microorganisms included *M. luteus* ATCC 9341, *E. coli* ATCC 13846, *B. subtilis* ATCC 6633, *S. marcescens*, *S. cerevisiae*, *C. albicans*, and *S. aureus* (clinical isolate and ATCC 25923). The clinical isolates were obtained as previously identified, anonymized archival strains from the culture collection of the Medical Microbiology Laboratory of the Kahramanmaras Sutcu Imam University Faculty of Medicine Hospital. These strains were chosen to represent Gram‐positive and Gram‐negative bacteria as well as yeasts commonly involved in food spoilage and human infections, and are widely used as reference organisms in plant extract antimicrobial screening. Bacterial and yeast strains were cultured on Mueller–Hinton and Sabouraud dextrose agar, respectively, bacterial suspensions were adjusted to a 0.5 McFarland standard (approximately 1.5 × 10^8^ CFU/mL), and yeast suspensions to a 0.5 McFarland standard (approximately 1 × 10^6^ CFU/mL). Extracts dissolved in 10% DMSO (16 mg/mL) were loaded into 6 mm wells (100 µL per well, corresponding to 1.6 mg extract per well); the plates were then kept at 4°C for 45 min to allow diffusion before incubation. Plates were incubated at 37°C for 24 h for bacteria and at 25°C for 48 h for yeasts. Inhibition zone diameters, measured with a caliper and including the 6 mm well, were recorded in millimeters, and the assay was performed in duplicate (two plates per organism). Ampicillin (10 µg) and nystatin (100 U) were used as positive controls for bacteria and fungi, respectively, and 10% DMSO served as the negative control. For extracts showing antimicrobial activity, MIC values were determined by the microdilution method in microwell plates. Extracts were serially diluted twofold in Mueller–Hinton broth (bacteria) or Sabouraud dextrose broth (yeasts), from 32 mg/mL down to 1 mg/mL, and each well was inoculated with 10 µL of a 0.5 McFarland suspension. A growth control (medium plus inoculum, without plant extract) was included and showed positive growth, and a solvent control (medium, inoculum, and 5% DMSO, the final in‐well solvent concentration) did not inhibit growth, confirming that the solvent did not contribute to the observed activity. After incubation, plates were read using a microplate reader (SPECTRAmax 384 PLUS) and the lowest concentration showing no visible growth was recorded as the MIC. All assays were conducted in triplicate.

### 4.8 Data Analysis and Visualization

All analytical measurements were performed in triplicate from the pooled composite samples. Results are expressed as mean ± standard error (SE), representing analytical repeatability rather than biological variation. Exploratory Pearson correlation analysis was conducted to evaluate the preliminary covariation between individual fatty acids and antioxidant parameters across the five sampling months (*n* = 5). The strength and direction of these descriptive relationships were visualized using correlation heatmaps. Antimicrobial activity results, obtained as inhibition zone diameters, were similarly visualized using heatmap‐based comparative analysis to demonstrate variation across sampling months and microbial species. All data handling and visualizations were performed using Python 3.10. The following libraries were used: NumPy and Pandas for data handling, SciPy for correlation coefficient calculations, and Seaborn and Matplotlib for graphical presentation.

## Author Contributions

**Fatma Dagli**: investigation, resources. **Nazan Comlekcioglu**: conceptualization, methodology, validation, formal analysis, data curation, writing – original draft, writing – review and editing, visualization, supervision, project administration. Both authors read and approved the final manuscript.

## Conflicts of Interest

The authors declare no conflicts of interest.

## Data Availability

The data that support the findings of this study are available from the corresponding author upon reasonable request.
